# Roles of medical, nursing and clinical specialists in selected African health systems: a document review of numbers, norms, training and scope of practices

**DOI:** 10.12688/wellcomeopenres.24053.2

**Published:** 2025-10-10

**Authors:** Seun Ajayi, Yingxi Zhao, Nada Ibrahim, Mike English

**Affiliations:** 1 NDM Centre for Global Health Research, Nuffield Department of Medicine, University of Oxford, Oxford, OX1 3SY, UK; 2 Jenner Institute, Nuffield Department of Medicine, University of Oxford, Oxford, England, OX3 7DQ, UK; 3 KEMRI-Wellcome Trust Research Programme, Nairobi, Nairobi County, Kenya

**Keywords:** Document analysis, education, task sharing, advanced nursing, clinical associate

## Abstract

**Background:**

Specialist health professionals are essential for meeting the evolving health needs of Sub-Saharan Africa (SSA), especially as the burden of complex and chronic conditions rises. They contribute not only to patient care but also to teaching, research, and policy development. However, there is a significant shortfall and uneven distribution of specialists across the region, creating major challenges for health systems. This paper examines the roles, numbers, training pathways, and scope of practice of medical, nursing, and clinical specialists in four SSA countries (Kenya, Uganda, South Africa, and Nigeria), with the aim of informing more effective workforce planning.

**Methods:**

Between September 2023 and July 2024, we conducted a document review of policies and guidelines related to specialist health professionals in Kenya, Uganda, Nigeria, and South Africa. Sources included ministries of health, regulatory bodies, academic institutions, and professional associations. We focused on the composition of the specialist workforce, training pathways, and defined roles across different health cadres.

**Results:**

There is marked variation in specialist workforce composition between countries. South Africa and Kenya reported the highest numbers of medical specialists (38% and 33% of all doctors respectively), while nursing specialists were more common in South Africa (40% of all nurses) and clinical officer specialist in Kenya (10% of all clinical officers). Training pathways ranged from university-based master’s programmes to national/regional fellowship systems. However, many curricula lacked essential non-clinical competencies - such as leadership, management, and communication skills - limiting specialists’ effectiveness in broader health system roles.

**Conclusion:**

Strengthening the specialist workforce in SSA requires better alignment between training and health system needs. This includes integrating non-clinical competencies into curricula, enhancing data systems for workforce planning, and addressing gaps in distribution and capacity. Policy reforms and strong leadership are critical to building a sustainable, well-equipped specialist workforce to meet the growing healthcare challenges.

## Plain English Summary

We looked at the number, roles, and training of specialist health professionals - such as doctors, nurses, and clinical officers - in four countries in Sub-Saharan Africa: Kenya, Uganda, Nigeria, and South Africa. The study was needed because many people in these countries now live with long-term or complex health problems, which require care from trained specialists. However, there are not enough specialists, and they are not evenly spread across the region. We reviewed documents from health ministries, professional associations, and universities between September and December 2023. We found that the types and numbers of specialists vary widely. South Africa and Kenya have more medical specialists, while Kenya and Uganda have more nurse and clinical officer specialists. We also found that specialist training programmes often do not include important non-clinical skills, such as leadership and communication. We recommended that better workforce planning is needed, including improved data systems and policies. A key limitation is that the findings are based on available documents from four English-speaking countries and may not fully reflect all specialist training programmes or the situation in other parts of Sub-Saharan Africa. The findings can help guide future planning to strengthen health systems in the region.

## Introduction

Human resources are a core component of the health system, and having adequate numbers in the right mix is vital for quality care and the delivery of Universal Health Coverage (UHC) ^1, 2^. The World Health Organisation (WHO) estimated that globally there were 65 million health workers in 2020 and an additional 19 million will be needed by 2030 ^3^. In Sub-Saharan Africa (SSA), over 70% of countries face critical shortages, with a projected gap of 6 million health workers by 2030 ^4^.

Demographic changes and epidemiological transitions also require countries to develop a health workforce to meet future needs ^5^. Non-communicable diseases are on the rise, child, adolescent and mental health needs are emerging concerns while maternal, neonatal, infectious diseases and emergency care remain pressing issues ^6^. Growing numbers of people with complex and chronic conditions increase the need for specialised, longitudinal and integrated care to optimise health. Specialist health professionals may then also be needed to improve health outcomes for patients with complex conditions while advancing teaching, research, and policy development ^7, 8^. For example, a recent synthesis of evidence from LMICs showed that medical specialists often make essential contributions not only to service delivery but also to governance and education ^9^.

However, there is a dearth of specialist health professionals in SSA. WHO estimated a total of 90,913 physician specialists in 2018 ^10^ with 0.7 million needed by 2030 ^11^. Data on other specialist workforce is not available from the WHO. There is limited or even absent capacity for specialist training programmes in some countries ^7^ and developing such training is complicated by the cost and duration of training. Specialist training also has historically and disproportionately focused on medicine, with two-thirds of specialist training programmes specifically for physicians in East and Southern Africa ^7^. There has been less policy discussion on balancing the training and development of medical, nursing and non-physician clinician specialists to produce the most cost-effective skill-mix ^12^. In addition, many countries with supply capacity often face distribution and retention challenges. Specialists are concentrated in urban cities and private facilities ^12^ creating a fragmentation in the referral system that threatens improvements in service availability and affordability. Initiatives such as the Consortium of Medical Schools in Africa provide opportunities at regional level to strengthen specialist education in ways that are more responsive to local needs ^13^. 

To better understand the development of specialist health professionals we examine four purposively-selected SSA countries (Kenya, Uganda, Nigeria, and South Africa). We focused on the three most common cadres of health professionals, i.e. medical, nursing and non-physician clinicians (e.g. clinical officers or associates). We reviewed their numbers, distributions, norms, training as well as their roles and duties in the health system.

## Methods

We generated and analysed data using the READ (Ready the materials, Extract data, Analyse data and Distil the findings) document review approach ^14^ to examine the role of medical, nursing and clinical officer specialists in African health systems. We report our methods and findings in line with the Checklist for the use and reporting of document analysis in health professions education research (CARDA) ^15^.

 We defined specialists as those with post-basic or post-entry-level qualifications, in line with the International Labour Organisation’s International Standard Classification of Occupations (ISCO-08) where medical specialists are defined as those with postgraduate training following a university undergraduate medical education ^16^. We specifically focused on four SSA countries, i.e. Kenya, Uganda, Nigeria, and South Africa, selected based on geographical context, the historical development of specialist training, and the availability of local contacts to sense-check findings.

For **readying the materials**, we conducted a systematic and sequential search of the websites of respective ministries of health, national regulatory bodies and designated professional bodies or associations in each country to identify documents on national health policies, HRH strategic plans, HRH norms and standards, relevant acts, schemes of service, scopes of practice, and task shifting and sharing policies. We also searched the websites of professional training institutions, universities, and fellowship-awarding colleges to assess core competencies in the curriculum and training on non-clinical skills. Due to time and resource availability, we could not engage with the websites of all training institutions and all specialities but focused searches on leading institutions, the four main medical specialisations (internal medicine, obstetrics and gynaecology, paediatrics and surgery) and prevailing specialities available per country for non-physicians. For example, in Kenya we sampled the University of Nairobi, which has the oldest University-based medical specialist training program, and in Nigeria the University of Nigeria, one of the earliest to introduce the recent National Universities Commission-approved postgraduate programmes in medicine ^17^. In addition, we searched databases of the WHO, World Bank, and the International Labour Organisation for relevant documents from those countries using similar keywords. We conducted the search between Sept 2023 and July 2024. We did not set any specific date or timeframe for including documents, and draft documents were also considered. We reviewed 71 documents and 9 websites. Documents gathered were archived for review and data extraction. All documents reviewed are publicly available and a list of documents reviewed is available in FigShare ^18^.

**Data extraction** was done using a customised Microsoft Word document. We specifically extracted the following content data: (1) the number of specialists, their distribution and speciality areas, (2) the training routes available, (3) the curriculum especially focusing on non-clinical contents such as leadership and management, (4) the staffing norms at different levels of the health system, and (5) the roles and responsibilities as listed in their scope of practices. We focused on the most recent data whereas possible. SA conducted the initial data extraction for the first two countries, and data were presented to YZ and ME to refine processes in line with the research question, before extractions were conducted for the two other countries. **Data analysis** was through an iterative process and emergent design with findings from each country informing further search and extraction in the other. We compared findings between speciality areas and cadres, and observed patterns within the country and also between countries. On **distilling**, we presented findings to a group of health policy and systems researchers based in the UK and the four studied countries who furnished further insights on the contexts and relevance. This step acted as a form of validation and triangulation, helping us check the consistency of interpretations against local expertise.

We acknowledge our own positionality as researchers in shaping this analysis. Three of the four authors are trained clinicians, two of whom are also health services researchers; the fourth author is a health systems and policy researcher with experience in document analysis. While two authors have direct work experience in Kenya and Nigeria, the team has ongoing collaborations with colleagues from Uganda and South Africa. Our embeddedness in these contexts informed both the identification and interpretation of documents.

## Results

Table 1 summarises the numbers, density, speciality areas, training pathways, and scope of practice of specialists across the four countries; the table is organised by cadre (column, medical, nursing, and clinical officer specialists) and country (row, Kenya, Uganda, South Africa, and Nigeria) to allow readers to compare patterns at a glance.

**Table 1.** Numbers, speciality areas, training pathways, staffing norms, and roles and responsibilities of medical, nursing and clinical specialists.

Country	Medical Specialists	Nursing and midwifery specialists	Clinical officer/associate specialists
**Kenya**	• **Number:** 4,908 out of 12,792 registered doctors in 2021 are specialists. Over 60% in the private sector. • **Density:** 0.42 specialists/10,000 population; 2.61 medical doctors/10,000 population • **Specialty areas:** In 2021, over 68% specialise OBGYN, general surgery, internal medicine, and paediatrics with OBGYN dominating. • **Training Pathways:** (1) University-based MMed; (2) ECSA Collegiate fellowship. • **Norms (2014):** 26 specialists spanning over 10 main specialties in level 4 or district hospitals • **Roles and Responsibilities:** New specialists work under supervision for 2 years before licensure and promotion require additional management and computer skills certification for managerial roles in facilities.	• **Number:** 109,659 total nurses and midwives in 2021. Over 80% in the public sector. In 2015, about 5.4% of registered nurses are specialists. • **Density:** 22.35 nurses/10,000 population • **Speciality areas:** In 2015, Over 50% are midwives; others include critical care nurses (13%), peri-operative nurses (7%), paediatric nurses (7%) and psychiatric nurses (6%). • **Training Pathways:** (1) Advanced diploma; (2) MSc in Nursing Science; (3) ECSA Collegiate Fellowship program. • **Norms (2014):** 43 specialist nurses in 9 different specialities at district hospitals • **Roles and Responsibilities:** Leadership roles in academia and PHC facilities and contribute to policy documents and guidelines.	• **Number:** 25,400 COs in 2021, with 62% in the private sector. In 2015, around 10% of COs have speciality training • **Density:** 5.18 clinical officers/10,000 population • **Speciality areas:** About 20 available speciality areas; Specialists available include Anaesthesia (29%), paediatrics (25%), ENT (12%) and lung & skin diseases (11%). • **Training Pathways:** (1) higher diploma (2) MSc • **Norms (2014):** 24 specialist CO at district hospitals • **Roles and Responsibilities:** Specialist COs can be deployed to head sub-county facilities or program units.
**Uganda**	• **Number:** 1,929 specialists out of 7,793 active doctors in 2022. Only 39% of doctors are in the public sector • **Density:** 0.43 specialists/10,000 population; 1.76 medical doctors/10,000 population • **Specialty areas:** In 2022, most common specialities are microbiology (17%), ob/gyn (16%), paediatrics (14%), and internal medicine (14%). • **Training Pathways:** (1) University-based MMed; (2) ECSA Collegiate fellowship. • **Norms (2023):** a specialist in the 4 main clinical areas at district hospitals • **Roles and Responsibilities:** Training and supervisory roles within the health system.	**Number:** Specialist nurses are 1,478 in number and include registered paediatric, palliative care, public health, and mental health nurses. There is a total 73,956 nurses and 32,959 midwives. • **Density:** 16.72 nurses and 7.46 midwives/10,000 population • **Training Pathways:** (1) Advanced diploma; (2) MSc in Nursing Science; (3) the ECSA collegiate fellowship program shared with 13 other Eastern and Southern African countries. • **Norms (2023):** 1 principal nursing officer (MSc required for this level) at district hospitals • **Roles and Responsibilities:** Plan, lead and coordinate nursing services	• **Number:** In 2022, out of 13,627 clinical officers, 264 specialised in Ophthalmology and 426 in ENT 426. Additionally, 345 specialised in Psychiatry and 250 in Anaesthesia. • **Density:** 3.08 clinical officers/10,000 population • **Training Pathways:** Diploma in Clinical Medicine, ENT and Neck Surgery, Mental Health and Ophthalmology. Additionally, those with a BSc can obtain an MPH, MSc in Health management or related studies for career advancement or civil service promotion • **Norms (2023):** 10 senior level COs with diploma-level specialisations at district hospitals • **Roles and Responsibilities:** only principal medical clinical officers’ role include research, supervision and management
**South Africa**	• **Number:** 14,199 specialists among 49,599 doctors in 2021, and the majority are in the private sector • **Density:** 1.00 specialists/10,000 population and 4.31 medical doctors/10,000 population (population covered by public healthcare) • **Speciality areas:** Most prevalent are anaesthesiologists, surgery, obstetricians/ gynaecologists and paediatricians • **Training Pathways:** MMed route together with Fellowship through the Colleges of Medicine of South Africa (CMSA) • **Norms (2012):** 4 specialists at district hospitals • **Roles and Responsibilities:** N/A	**Number:** In 2022, about 110,000 of 271,431 nurses registered with SANC have additional qualifications but speciality status could not be ascertained. • **Density:** 14.80 professional nurses/10,000 population and 28.18 nurses/10,000 population (population covered by public healthcare) • **Speciality areas:** In 2022, about 40% specialised in nursing administration and almost 30% in nursing education • **Training Pathways:** (1) postgraduate diploma (2) MSc • **Norms (2012):** At least 3 specialists at district hospitals • **Roles and Responsibilities** Specialists head facilities and audit the quality of nursing and healthcare	**Number:** As of 2021, 1,089 Clinical Associates are registered with the Health Professions Council of South Africa (HPCSA). No speciality qualifications. • **Density:** 0.08 clinical associates/10,000 population • **Speciality areas:** N/A • **Training Pathways:** University-based honours in Emergency Medicine • **Norms :** N/A • **Roles and Responsibilities:** supervised for a period of 5 years before clearance to work independently. First 2 years under a Medical Practitioner. Those with experience between 2 and 4 years may be supervised by an experienced fellow clinical associate.
**Nigeria**	• **Number:** 9,364 of 84,277 doctors in 2020 are specialists. • **Density:** 3.86 medical doctors/10,000 population • **Speciality areas:** About 20 main specialities areas available through the training pathways combined • **Training Pathways:** (1) National Postgraduate Medical College; (2) West African colleges; (3) MSc in Medicine • **Norms :** N/A • **Roles and Responsibilities:** N/A	• **Number:** There are 333,657 nurses and midwives in 2020, stock of specialists could not be ascertained. • **Density:** 15.6 nurses and midwives/10,000 population • **Speciality areas:** N/A • **Training Pathways:** (1) Postgraduate diploma (2) university-based MSc route (3) West African Collegiate fellowship. • **Norms :** N/A • **Roles and Responsibilities:** N/A	There is no clinical officer/associate program in Nigeria.

Note: Across the four countries, specialists are required at the First Referral Hospitals (FRHs) and not below. In this context, the FRH are primary hospitals at the interface between primary healthcare clinics and the advanced specialist or tertiary hospitals. They are level 4 / county hospitals in Kenya, General Hospitals in Uganda, District hospitals in South Africa and commonly General or District hospitals in Nigeria. They provide in-patient and outpatient services including ob-gyn, paediatric, internal medicine and general surgical services. Depending on the size, they have 50 to 600 beds and cater for about 100,000 to 500,000 population

### Doctors

Exploring medical specialists in Kenya, Uganda, South Africa, and Nigeria reveals notable differences in the composition and specialisation trends ( Table 1). Available speciality training areas range from 10 to 29 and with varying numbers of sub-specialities ^19– 22^. In 2020/2021, 38% of doctors in Kenya are reported to be specialists, South Africa and Uganda have slightly lower proportions at 33% and 25% ^20, 23^, while Nigeria, with the highest absolute number of doctors, reports only 11% as specialists ^24^. In Kenya, the four main clinical hospital disciplines are the most common with the highest number of specialists in obstetrics and gynaecology (19%) ^25^. In comparison, there is a higher proportion of specialists in microbiology in Uganda (17%), and surgery (11%) and anaesthesiology in South Africa (14%) ^26, 27^. Across these countries, most specialists are within the private sector.

Training pathways for medical specialisation differ spanning the university-based Master of Medicine (MMed) program, regional or national collegiate training and fellowship routes. While the MMed program has existed in East and South Africa, it was recently introduced by the Nigeria Universities Commission ^17^. These university-linked MMed programmes are often a blend of classroom and hospital rotations over 2 to 6 years depending on the speciality area. In comparison, most collegiate fellowships are hospital-based. The East Central and Southern Africa College of Health Sciences (ECSA-CHS), a regional college of the region’s member states, serves Kenya and Uganda. South Africa operates an independent national collegiate system i.e. Colleges of Medicine of South Africa. Nigeria belongs to the West African Colleges of Surgeons and Physicians but also operates another national fellowship program through its National Postgraduate Medical College.

In all four countries, at least a limited range of medical specialists are required at the first referral hospital level i.e. district level, but not below. The referral system and approach to staffing medical specialists in health facilities differ per available norms and standards. Uganda recommends a specialist in each of the four major clinical areas at the district level ^28^, and, also at this level, South Africa proposes a skill mix of a paediatrician, gynaecologist, family physician and anaesthesiologist ^29^. On the other hand, the approach in Kenya is more ambitious, recommending that there be 24 specialists spanning 17 speciality areas in its level 4 county hospitals ^30^. Apparent differences may also reflect the expected population catchment areas of these different hospitals.

Aside from their core clinical service responsibilities, medical specialists are often also expected to have departmental or even facility-level hospital leadership and management roles. However, our document review found little evidence of modules to build capacity for these roles in training curricula. In some settings, however, on-the-job training and certification in strategic leadership and computer skills are essential for career promotion ^31^.

### Nurses and midwives

The number of areas available for nursing and midwifery specialists varies from country to country but the specialist stock was not specified in most databases and documents reviewed especially for Uganda and Nigeria. In Kenya, reportedly 5.4% of its 70,000 registered nursing workforce in 2020 are specialists, over half of whom are midwives ^32, 33^. About 40% of nurses and midwives registered with the South African Nursing Council (SANC) in 2022 were reported to have additional qualifications, levels of which were not specified ^34^. However, about 80% of new specialists registered by SANC for additional qualifications in 2022 were in nursing administration, nursing education and occupational health nursing ^35^.

The specialist nursing training in Uganda and Kenya is offered through the university-led advanced diploma, Master of Nursing Science programs or a collegiate fellowship through the ECSA-CHS ^36^. While there are many speciality areas offered through MSc training, those through the ECSA-CHS are limited to fellowships in chronic disease management, critical care, midwifery and neonatal care. Similarly in Nigeria nurse speciality training is offered through a postgraduate diploma, an MSc or the West African Collegiate fellowship – especially for those already with a MSc and interested in research ^37^. Specialist training in South Africa could be obtained either through a postgraduate diploma or an MSc route ^38^. In contrast to medical speciality training most nursing speciality training curricula assessed involve a blend of facility and community postings and have health system strengthening, leadership and communications assessed as core competencies.

In terms of staffing norms, a minimum of 43 specialist nurses across nine speciality areas (midwives specialists excluded) are recommended in norms for level 4 hospitals in Kenya ^30^ while at the district level Uganda and South Africa recommend one and three specialist nurses ^28^ respectively, with those in South Africa including a midwife, paediatric nurse and a specialist Primary Health Care Nurse. The latter is a cadre introduced in South Africa to provide a continuum of care, supervise nursing services and manage facilities within the district ^29, 39^. No standards or norms were identified for general or district hospitals in Nigeria. According to the available scope of practice in different countries, specialist nurses are expected to play pivotal roles in leading health programs, coordinating health services at lower-level healthcare facilities and auditing the quality of care. Similar to medical doctors, career progression in some quarters requires additional leadership training and hands-on skills like computer use.

### Clinical officers/associates

The clinical officer workforce exists in three countries studied i.e., Kenya, Uganda and South Africa (clinical associates), but the cadre is yet to be introduced in Nigeria. Kenya has the most varied clinical officer workforce, with 10% having speciality training across 20 speciality areas ^40, 41^, most commonly in anaesthesia (29%) and paediatrics (25%) ^32^. Nearly two-thirds of Kenyan clinical officers are in the private sector ^23^. Over 13,600 COs have been registered in Uganda but the number of those with specialist qualifications could not be ascertained ^42^. In South Africa, clinical associates were developed relatively recently and a total of 1,089 are registered with the Health Professions Council of South Africa (HPCSA) and no formal specialist qualification exists, other than a short honours year in emergency medicine ^43^.

Similar to nursing training, specialised CO training is available through the diploma and MSc routes. In Kenya, diploma trainees can proceed to a specialised 2-year higher national diploma program at medical training colleges, while there is also an MSc program through some universities for those with undergraduate training. Findings from Uganda show diploma-level training in clinical medicine, ENT, psychiatry and ophthalmology. In addition, those with a bachelor's degree can obtain a Master’s in public health or health management for career progression.

The norms and standards provided for 24 specialist clinical officers in 10 specialities in Kenyan level 4 hospitals ^30, 32^. The Kenyan scheme of service for specialist clinical officers indicates they can be deployed to head lower-level facilities, coordinate referrals and oversee health programmes ^44^. This is similar to a Principal clinical officer, a leadership type role and the career peak in Uganda who is expected to hold a MSc degree ^45^. As far as we could ascertain most specialist clinical officer training curricula also lack a leadership component to prepare trainees for these roles.

## Discussion

Our study sought to explore the number, distribution, training as well as roles and responsibilities of the specialist health workforce in SSA. We found that while specialist medical and nursing roles exist in all four countries studied, only Kenya and Uganda have specialist clinical officers. Across all the countries, the number of specialists is limited and they are unevenly distributed, with most working in private settings and urban cities. This contrasts at least in Kenya with relatively high specialist staffing norms for all three main cadres at level 4 or district hospitals. Opportunities for specialisation differ between countries and cadres. Training routes for specialists are similar and mixed in all countries and across cadres through either university-led masters or advanced diploma courses, or national and regional collegiate fellowship systems. While all three specialist cadres’ roles and responsibilities include clinical care, supervision and management including heading health facilities, their training curricula do not necessarily cover these non-clinical skills to prepare them for these roles.

In recent years, specialist training has expanded in LMICs and SSAs, primarily fueled by individual preference for advanced education, rising incomes, medical tourism, and market dynamics, with minimal influence from policy interventions ^8^. For example, it was reported in 2021 that there are 159 medical speciality and subspecialty programs in Kenya, 74 in South Africa and 15 in Uganda ^7^. The number of nursing speciality and subspecialty programs in these three countries added up to 31 ^7^. As the development of specialist training proceeds, there is a growing number of trained generalist health workers but also emerging unemployment and underemployment problems, thus specialist training may seem an attractive option to secure a career ^11, 46^. These factors persist in influencing the choice of specialisations, resulting in an oversupply in certain areas and the creation of new (sub)-specialities that may not provide the necessary skill mix to meet the services required by the population ^47, 48^. Rather than intentional policies, such dynamics often are shaped by market forces ^8^ with a resulting mismatch of supply and demand which could lead to a variety of challenges including workforce dissatisfaction and outmigration. 

The significant country differences in the types and numbers of specialities highlight the ongoing evolution of task-sharing across different healthcare roles. For instance, in South Africa, anaesthesia is the most common speciality for doctors, while in Kenya, it is more popular among clinical officers. In Tanzania, the assistant medical officer role has been developed to specifically share emergency surgical obstetric services with doctors ^49^. These differences reflect not only the varying levels of task-sharing within specific specialities but also the professional jurisdictions that may resist such changes, particularly from established medical bodies ^8^. For example, South Africa’s clinical associates are still without a specialisation qualification and are regulated under the Medical and Dental Professionals Board. Moreover, as specialists often take on leadership and management roles, the lack of preparation in these areas, as highlighted in studies on specialist clinical officers, underscores the need for curricula that integrate clinical leadership, management, communication, and quality improvement alongside technical training ^50, 51^. 

With demographic and epidemiological transitions, changing population health demand, more speciality and sub-speciality training programmes offered through different training pathways all influencing specialist workforce supply and demand, we believe the urgent policy question countries need to ask is “What type of specialists will countries need, and taking up what roles?”. As the configuration of health systems and UHC differs across countries ^52^, there is no simple answer to this question. Countries like Kenya previously set relatively high specialist staffing norms for first referral hospitals recommending 26 medical specialists spanning 10 main specialities, 43 specialist nurses in 9 specialities and 24 specialist clinical officers. Such norms have not been achieved. Even in relatively well-staffed internship training hospitals less than half had four or more medical specialists, and the financial cost associated with meeting the recommended norms would be impossible to achieve for many years ^53, 54^. Currently, the majority of specialist services especially in first referral level hospitals are either not delivered or are delivered by general medical doctors (including interns) together with generalist nurses and clinical officers ^55^. How different skill-mix patterns, between generalists and specialists, between different cadres (medical doctors, nurses and clinical officers) affect service delivery and outcomes has rarely been studied ^47, 56^.

This study has several key policy implications. First, governments need to provide clearer leadership and stronger policy direction, in collaboration from multiple stakeholders, to align specialist training with public sector needs and ensure a sustainable workforce ^57^. Second, countries would benefit from more robust human resource management information systems to monitor the supply, distribution, and employment of all specialist cadres. In particular, the absence of an ISCO-08 code to for non-physician specialists limits reporting and planning (whereas medical professionals have separate codes for generalists and specialists); developing such codes would facilitate more accurate workforce tracking. Third, as specialists often take on leadership and management roles but felt unprepared for them, training programmes should integrate non-clinical competencies such as management, communication, and quality improvement into curricula.

Several limitations should be considered while interpreting our findings. We are limited to only four, notably all anglophone SSA countries, purposively selected based on geographical and historical context, as well as the availability of local contacts to sense-check findings. Within each country, despite trying to be comprehensive in our search of the documents reviewed, we are unable to, for example, search all the training institutions and all speciality training programmes they offer. Some documents reviewed are regional level and may not reflect what is obtained at the national level. In addition, while we reviewed the distribution of specialists between public and private or faith-based sectors, our focus is primarily on specialists’ role in public health systems, considering in many SSAs the majority of hospital services for the poor are still provided through this sector. Last but not least, there are often variations and discrepancies between different data sources despite all of them claiming to represent the same indicator, therefore there is a need for improved workforce data collection and collation.

## Conclusion

The specialist workforce is crucial to mitigating the demographic and epidemiological transition and increasing complex and chronic service needs in SSAs. These workforces are however limited and unevenly distributed with a concentration in the private sector and urban areas. Their production is often not aligned with health systems needs, nor is their training curricula aligned with their scope of practices. Policymakers, regulatory bodies and training institutions therefore need to re-consider the number, type and role of the specialist workforce, especially skill mixes between different qualifications, disciplines and experiences. Priorities should include stronger workforce planning, improved workforce data systems, and integrating non-clinical competencies into training to ensure a sustainable and effective specialist workforce.

## Abbreviations

CO, clinical officer

ECSA-CHS, East Central and Southern Africa College of Health Sciences

HPCSA, Health Professions Council of South Africa

HRH, human resources for health

ISCO, International Standard Classification of Occupations

LMICs, low- and middle-income countries

MMed, Masters of Medicine

SANC, South African Nursing Council

SSA, Sub-Saharan Africa

WHO, World Health Organization

UHC, Universal Health Coverage

## Declarations

### Ethics and consent

Ethical approval and consent were not required.

### Consent for publication

Not required.

## Data Availability

No primary data are associated with this study. All documents analysed were publicly available at the time of the review and are fully cited within the article and/or appendix. Figshare: Roles of medical, nursing and clinical specialists in selected African health systems: a document review of numbers, norms, training and scope of practices. https://doi.org/10.6084/m9.figshare.28678544.v1 ^18^. This project contains the following extended data: Appendix Data are available under the terms of the Creative Commons Attribution 4.0 International license (CC-BY 4.0).
