# Supplementary material for: Roles of medical, nursing and clinical specialists in selected African health systems: a document review of numbers, norms, training and scope of practices
Source: Wellcome Open Res. 2025 Oct 10;10:313. Originally published 2025 Jun 9. [Version 2] doi: 10.12688/wellcomeopenres.24053.2 (PMC12592852; doi:10.12688/wellcomeopenres.24053.2)
Supplement: Peer review history

# Reviewer report: Reviewer response for version 2

**DOI:** 10.21956/wellcomeopenres.27648.r135736

**Published:** 2025-11-06

**Authors:** Abba Amsami Elgujja

**Affiliations:** 1 Al-Ansr University, MAIDUGURI, Borno, Nigeria

I have thoroughly reviewed this version, and found significant improvements as compared to the other versions, and it has substantially complied with my recommendations.  I have no reservations in allowing the manuscript to proceed with indexing in its current form. Thank you

Is the work clearly and accurately presented and does it cite the current literature?

Partly

If applicable, is the statistical analysis and its interpretation appropriate?

I cannot comment. A qualified statistician is required.

Are all the source data underlying the results available to ensure full reproducibility?

Partly

Is the study design appropriate and is the work technically sound?

Partly

Are the conclusions drawn adequately supported by the results?

Partly

Are sufficient details of methods and analysis provided to allow replication by others?

Partly

Reviewer Expertise:

NA

I confirm that I have read this submission and believe that I have an appropriate level of expertise to confirm that it is of an acceptable scientific standard.

# Reviewer report: Reviewer response for version 2

**DOI:** 10.21956/wellcomeopenres.27648.r135737

**Published:** 2025-10-31

**Authors:** Luís Furtado

**Affiliations:** 1 University of the Azores, Angra do Heroísmo, Portugal

No further comments.

Is the work clearly and accurately presented and does it cite the current literature?

Yes

If applicable, is the statistical analysis and its interpretation appropriate?

Not applicable

Are all the source data underlying the results available to ensure full reproducibility?

Yes

Is the study design appropriate and is the work technically sound?

Yes

Are the conclusions drawn adequately supported by the results?

Yes

Are sufficient details of methods and analysis provided to allow replication by others?

Yes

Reviewer Expertise:

Evidênce-based practice; Literature review; Epidemiology, Quantitative research

I confirm that I have read this submission and believe that I have an appropriate level of expertise to confirm that it is of an acceptable scientific standard.

# Reviewer report: Reviewer response for version 1

**DOI:** 10.21956/wellcomeopenres.26537.r131739

**Published:** 2025-09-08

**Authors:** Abba Amsami Elgujja

**Affiliations:** 1 Al-Ansr University, MAIDUGURI, Borno, Nigeria

Dear Editor,

 Thank you for the opportunity to review this important manuscript. The paper tackles a critical and timely topic — the availability, training, and roles of specialist health professionals in Sub-Saharan Africa. With the growing burden of complex health needs in the region, this work has strong potential to guide both policy and practice.

**Strengths:**

 The title is clear and informative, and the abstract, along with the Plain English Summary, makes the study accessible to a wide audience. The introduction provides a solid context, drawing on WHO data to highlight workforce challenges. The methods are systematic and transparent, and the results offer valuable insights into gaps such as the lack of non-clinical skills in training programs. Overall, the paper is well-structured, and the references are comprehensive.

**Areas for Improvement:**

 Some revisions are needed to strengthen the manuscript’s clarity and impact. The abstract would benefit from including the names of the four countries studied and a few key statistics to give readers a clearer picture. While the introduction sets the stage well, it could engage more deeply with existing literature and explain more clearly why these specific countries were chosen. The methods, though systematic, rely heavily on publicly available documents without any quality checks or triangulation, which raises concerns about reliability.

 The results are rich, but at times overwhelming. Table 1, in particular, is very dense and could be split into smaller tables or supported by visuals such as charts or graphs to improve readability. The discussion highlights important issues like workforce governance and task-shifting but remains mostly descriptive and could offer more concrete, actionable recommendations. Similarly, the conclusion reiterates the findings but does not provide clear next steps for policymakers.

**Overall Recommendation:**

 This is a valuable and much-needed study. With some revisions to strengthen the abstract, expand the framing of the introduction, improve how data are presented, and provide more forward-looking recommendations, the manuscript will be well-positioned to make a strong contribution to the field.

 I recommend **minor to moderate revisions** and encourage the authors to refine these areas so the paper can reach its full potential.

 Kind regards,

Is the work clearly and accurately presented and does it cite the current literature?

Partly

If applicable, is the statistical analysis and its interpretation appropriate?

I cannot comment. A qualified statistician is required.

Are all the source data underlying the results available to ensure full reproducibility?

Partly

Is the study design appropriate and is the work technically sound?

Partly

Are the conclusions drawn adequately supported by the results?

Partly

Are sufficient details of methods and analysis provided to allow replication by others?

Partly

Reviewer Expertise:

Healthcare law, infection prevention and control, digital health governance, AI ethics in healthcare, and health workforce policy.

I confirm that I have read this submission and believe that I have an appropriate level of expertise to confirm that it is of an acceptable scientific standard, however I have significant reservations, as outlined above.

# Response

**Published:** 2025-10-03

**Authors:** Yingxi Zhao

**Affiliations:** University of Oxford Nuffield Department of Medicine, Oxford, England, UK

Thank you for your constructive feedbacks, we have made several changes to strengthen the clarity, depth and policy relevance of this paper.  

1. The abstract would benefit from including the names of the four countries studied and a few key statistics to give readers a clearer picture.

We agree with this comment and have revised the abstract to add the names of the four study countries (Kenya, Uganda, Nigeria, and South Africa), and included key statistics on specialist workforce numbers in these countries.  

1. While the introduction sets the stage well, it could engage more deeply with existing literature and explain more clearly why these specific countries were chosen.

We have strengthened the introduction but citing two new references, including a systematic review of medical specialists in LMICs (Russo et al. 2025) and a commentary on the Consortium of Medical Schools in Africa (Bekele et al. 2025). In the methods sections (2 ^nd^ paragraph), we clarified that the four study countries were purposively chosen based on geographical context, the historical development of specialist training, and the availability of local contacts to sense-check findings.  

1. The methods, though systematic, rely heavily on publicly available documents without any quality checks or triangulation, which raises concerns about reliability.

We now state clearly in the methods that findings were checked by local researchers where the authors were not affiliated, and also presented to a group of health policy and systems researchers based in the UK and the four studied countries. This process furnished further insights on the contexts and relevance. We added a sentence noting that this step acted as a form of validation and triangulation, helping us check the consistency of interpretations against local expertise.  

1. The results are rich, but at times overwhelming. Table 1, in particular, is very dense and could be split into smaller tables or supported by visuals such as charts or graphs to improve readability.

We thank the reviewer for this thoughtful suggestion. We carefully considered whether splitting Table 1 would improve clarity; however, given that Reviewer 1 specifically noted that Table 1 is “well-structured” and provides a “clear summary of the core findings,” and that splitting this into 3 tables by medical specialist, nursing specialist and clinical officer specialist won’t necessarily improve readability, we decided to retain it in its current format. We agree that readability is important, and to address this, we have added a short explanatory sentence in the results section to guide readers on how to navigate the table. We believe this strikes a balance between maintaining the integrity of the table, as positively recognised by Reviewer 1, and responding to Reviewer 2’s concern about readability.  

1. The discussion highlights important issues like workforce governance and task-shifting but remains mostly descriptive and could offer more concrete, actionable recommendations. Similarly, the conclusion reiterates the findings but does not provide clear next steps for policymakers.

We have addressed this by adding a new paragraph at the end of the Discussion that sets out three clear policy implications arising from our findings, including stronger government leadership, more robust human resource data system, and integration of non-clinical competencies into training. In the conclusion, we now explicitly reintegrate these next steps, providing clearer next steps for policy papers.

# Reviewer report: Reviewer response for version 1

**DOI:** 10.21956/wellcomeopenres.26537.r125497

**Published:** 2025-06-24

**Authors:** Luís Furtado

**Affiliations:** 1 University of the Azores, Angra do Heroísmo, Portugal

I would like to express my gratitude for the opportunity to review this document review study. It is my view that studies of this nature are essential in providing valid, relevant evidence and practical tools for healthcare policymakers, particularly in the domains of health workforce planning and training policy.

**Overall Assessment**

 The manuscript is clearly written, well-structured, and highly relevant to the topic under investigation. The writing is careful and coherent, and the structure is logically aligned with the aims and nature of a document review.

**Introduction**

 The introduction is clear and offers a solid conceptual and theoretical grounding for the study. I have no suggestions for improvement in this section.

**Methods**

 The Methods section is detailed, well-organised, and coherent, allowing readers to assess the rigour and reliability of the document review. It adequately outlines the review process and lends credibility to the study’s methodological foundations.

**Results**

 The Results section is very well written. It presents the findings from the document analysis with clarity and precision, without becoming overly lengthy or repetitive. The analysis is systematically reported for each professional category and further disaggregated by country and type of healthcare infrastructure. The section is highly readable and accessible. Table 1 presents a well-structured summary of the core findings arising from the document analysis and synthesis.

**Discussion**

 The discussion is highly pertinent and insightful. It raises key issues concerning future policy options across countries and healthcare system levels. The authors succeed in prompting reflection on the implications of different approaches to structuring and deploying highly specialised—and often scarce—human resources for health.

 The paragraph addressing the study’s limitations is appropriately detailed and reinforces the trustworthiness of the article. It demonstrates the authors’ commitment to scientific integrity by acknowledging the inherent constraints of their work.

**Conclusion**

 No comments. The conclusions are appropriate and well aligned with the findings and scope of the study.

**References**

 The references cited are relevant and appropriate to the study. They appear up to date, further underscoring the timeliness of the topic addressed.

**Recommendation**

 In light of the above, I recommend the acceptance of this manuscript for indexing.

Is the work clearly and accurately presented and does it cite the current literature?

Yes

If applicable, is the statistical analysis and its interpretation appropriate?

Not applicable

Are all the source data underlying the results available to ensure full reproducibility?

Yes

Is the study design appropriate and is the work technically sound?

Yes

Are the conclusions drawn adequately supported by the results?

Yes

Are sufficient details of methods and analysis provided to allow replication by others?

Yes

Reviewer Expertise:

Evidênce-based practice; Literature review; Epidemiology, Quantitative research

I confirm that I have read this submission and believe that I have an appropriate level of expertise to confirm that it is of an acceptable scientific standard.
